# Biomarkers of Response to Internet-Based Psychological Interventions: Systematic Review

**DOI:** 10.2196/55736

**Published:** 2024-11-29

**Authors:** Giulia Gotti, Chiara Gabelli, Sophia Russotto, Fabio Madeddu, Philippe Courtet, Jorge Lopez-Castroman, Patrizia Zeppegno, Carla Maria Gramaglia, Raffaella Calati

**Affiliations:** 1 Department of Psychology University of Milan-Bicocca Milan Italy; 2 Department of Translational Medicine University of Eastern Piedmont Novara Italy; 3 PSNREC, University of Montpellier INSERM Centre Hospitalier Universitaire de Montpellier Montpellier France; 4 Department of Emergency Psychiatry and Acute Care Lapeyronie Hospital Centre Hospitalier Universitaire de Montpellier Montpellier France; 5 FondaMental Fondation Créteil France; 6 Centro de Investigación Biomédica en Red de Salud Mental Madrid Spain; 7 Department of Adult Psychiatry Nimes University Hospital Nimes France; 8 Department of Psychiatry, Radiology, Public Health, Nursing and Medicine University of Santiago de Compostela Santiago de Compostela Spain; 9 Institute of Psychiatry University of Piemonte Orientale Novara Italy; 10 Struttura Complessa Psichiatria Azienda Ospedaliero-Universitaria Maggiore della Carità Novara Italy

**Keywords:** biomarker, cognitive behavioral therapy, internet-based intervention, systematic review, psychological intervention, mental health intervention, meta analysis, psychiatric, blood glucose, mindfulness, stress management, immune response, smoking, cortisol

## Abstract

**Background:**

Internet-based psychological interventions provide accessible care to a wide range of users, overcoming some obstacles—such as distance, costs, and safety—that might discourage seeking help for mental issues. It is well known that psychological treatments and programs affect the body, as well as the mind, producing physiological changes that ought to be considered when assessing the efficacy of the intervention. However, the literature investigating changes in biomarkers specifically after internet-based psychological and mental health interventions has not yet extensively inquired into this topic.

**Objective:**

This systematic review aims to provide a synthesis of literature examining the effects of internet-based psychological interventions—targeting both clinical (mental and physical) and nonclinical conditions—on biomarkers. A secondary aim was to evaluate whether the biomarkers’ variations were related to a complementary modification of the psychological or physical symptoms or to a general improvement of the participants’ well-being.

**Methods:**

This review was conducted according to the PRISMA (Preferred Reporting Items for Systematic Reviews and Meta-Analysis) statement. A literature search was performed through 3 databases (PubMed, PsycINFO, and Scopus). Studies examining changes in biomarkers before and after internet-based psychological interventions or programs targeting both clinical and nonclinical samples were included, with no exclusion criteria concerning mental or physical conditions.

**Results:**

A total of 24 studies fulfilled the inclusion criteria. These studies involved individuals with psychiatric or psychological problems (n=6, 25%), those with organic or medical diseases (n=10, 42%), and nonclinical populations (n=8, 33%). Concerning psychiatric or psychological problems, cognitive behavioral therapy (CBT) and CBT-informed interventions showed partial effectiveness in decreasing glycated hemoglobin blood glucose level (n=1) and chemokines (n=1) and in increasing connectivity between the default-mode network and the premotor or dorsolateral prefrontal cortex (n=1). Among individuals with organic or medical diseases, studies reported a significant change in cardiac or cardiovascular (n=3), inflammatory (n=2), cortisol (n=2), glycated hemoglobin (n=2), and immune response (n=1) biomarkers after CBT and CBT-informed interventions, and mindfulness and stress management interventions. Lastly, mindfulness, CBT and CBT-informed interventions, and music therapy succeeded in modifying immune response (n=2), cortisol (n=1), α amylase (n=1), posterior cingulate cortex reactivity to smoking cues (n=1), and carbon monoxide (n=1) levels in nonclinical populations. In some of the included studies (n=5), the psychological intervention or program also produced an improvement of the mental or physical condition of the participants or of their general well-being, alongside significant variations in biomarkers; CBT and CBT-informed interventions proved effective in reducing both psychological (n=2) and physical symptoms (n=2), while a mindfulness program successfully lowered cigarette consumption in a nonclinical sample (n=1).

**Conclusions:**

Although further evidence is required, we hope to raise awareness on the potential impact of internet-based interventions on biomarkers related to mental and physical health.

## Introduction

### Internet-Based Interventions

In the psychological and mental health field, a growing body of evidence has demonstrated telepsychiatry’s ability to provide accessible, wide-ranging, and high-quality services. This allows us to overcome obstacles, such as distance, costs, and safety, making these methods preferable for users who live afar, have transportation difficulties, or have time limitations. It also fosters a sense of empowerment [1]. This explains that online approaches to psychological therapy might be more easily accepted than standard psychotherapy. Additionally, an online setting might create in the users an apparent sense of anonymity, promoting feelings of safety and self-disclosure and reducing the stigma typically associated with seeking mental health support [2-4]. Internet-based programs exhibit remarkable versatility, providing a diverse array of services to a broad spectrum of users. This includes both patients and healthy individuals across various age groups, as well as specific populations such as health care professionals [1].

On the other hand, online interventions might also carry some negative issues: one of the main disadvantages is the risk of potential breaches of privacy, confidentiality, and data security, likely due to the use of unsecured websites or easily hackable software [4]. They may also entail some inconveniences for both users and health care professionals and institutions, such as the need for adequate technological equipment and systems compatibility. Accordingly, cost-effectiveness is a relevant issue, and results from research on this topic are mixed, bringing evidence of both methodological flaws and similar—or in some cases, better—cost-effectiveness compared to traditional in-person therapy [1,4]. Furthermore, the remote nature of online interventions themselves leads to a higher risk of low adherence and greater dropout rates [3,4].

### Biomarkers

According to the National Institutes of Health (NIH), a biomarker is “a defined characteristic that is measured as an indicator of normal biological processes, pathogenic processes, or biological responses to an exposure or intervention, including therapeutic interventions. Biomarkers may include molecular, histologic, radiographic, or physiologic characteristics” [5]. The US Food and Drug Administration (FDA)–NIH Biomarker Working Group defined a taxonomy of biomarkers, distinguishing diagnostic, monitoring, response, predictive, prognostic, safety, and susceptibility or risk biomarkers [6]. Response biomarkers, in particular, are used to evaluate change in a biological response, which can potentially be beneficial or harmful, in an individual after being exposed to a medical condition, a clinical intervention (including drug treatments), or an environmental agent.

Within therapeutic interventions, examining potential variations in biomarker levels before and after treatment proves to be an effective method for objectively assessing the treatment’s effectiveness. Additionally, it facilitates a deeper understanding of whether these biological modifications align with concurrent changes in the symptoms targeted by the intervention. Mental and physical health are strictly linked and they profoundly impact one another [7]. Thus, psychological treatments and programs can also affect the body, as well as the mind, with beneficial effects on stress, the immune system, and brain activity [8].

A vast body of literature shows changes in biomarkers following psychological interventions. For example, psychological interventions, such as cognitive behavioral therapy (CBT), consistently proved to be efficient in improving both the immune system [9] and neurological functioning biomarkers [10,11] across various mental and organic health conditions. Moreover, Claudino et al [10] found biomarker variations to be associated with symptoms reduction, allowing authors to consider them as a reliable way to assess treatment response [9]. Finally, similar results were reported not only for standard psychological interventions but also for various psychosocial programs, such as music-based psychosocial intervention, together with reductions in cortisol and inflammatory activity [12].

Nonetheless, there is scarce evidence of the efficacy of internet-based psychological interventions in changing biomarkers across different health conditions. Considering the benefits of online treatments and the rapid spreading of technologies in health care—especially following the COVID-19 pandemic [13]—a systematic review is needed to comprehensively synthesize the current state of research, and it will help assess the efficacy of internet-based programs, compared to in-person ones.

### Aim

The main objective of this systematic review was to investigate the effects of internet-based psychological interventions targeting every type of condition (mental or physical) on various biomarkers, assessed before and after the intervention itself. Additionally, we aimed to determine whether the biomarkers’ variations were related to a complementary modification of the underlying mental or medical health condition, if present, or of the nonclinical issue addressed by the intervention or program.

The literature investigating changes in biomarkers after internet-based psychological and mental health interventions has not yet extensively inquired into this topic, and to the best of our knowledge, this is the first systematic review on this topic.

## Methods

### Procedure

The review was not registered. This systematic review was performed according to the PRISMA (Preferred Reporting Items for Systematic Reviews and Meta-Analysis) statement [14]. A literature search (on articles published until May 25, 2023) was independently performed by GG, CG, SR, and CMG using the PubMed, PsycINFO, and Scopus databases, with the search terms reported in Multimedia Appendix 1. The reference lists of the identified meta-analysis and reviews were also checked to find additional relevant articles. GG, CG, and SR independently evaluated the suitability of all the identified papers; in case of conflicting opinions regarding the inclusion or exclusion of a paper, GG, CG, and SR consulted with each other and with RC to find an agreement.

We included studies that investigated internet-based or app-based, remote, psychological or psychoeducational interventions or programs and that measured biomarkers before and after the intervention or program. Studies were included if:

1. They administered a psychological or psychoeducational intervention, or a multicomponent intervention based on a psychological approach.
2. At least half of the intervention or program had to be provided online or through a mobile phone app so that it could take place at the participant’s home.
3. Biomarkers were assessed both before and after the intervention or program.
4. They targeted psychiatric, psychological, or physical conditions or nonclinical individuals (we considered “nonclinical” as those participants who were not recruited based on a specific psychiatric or organic illness and those interventions that did not specifically target a certain disease—in opposition to “psychiatric or psychological” and “physical” samples, whose participants had a mental or medical diagnosis).
5. They were written in English, French, or Italian.

Studies were excluded if:

1. They assessed biomarkers only before or only after the intervention or program.
2. They provided an internet-based psychological intervention or program without assessing any biomarker (eg, the study by ter Huurne et al [15]).
3. The intervention or program took place entirely in a prearranged setting, specifically ideated for this study, such as in-person, virtual reality (eg, the study by Yang et al [16]), and computer-assisted interventions.
4. They involved lifestyle interventions or programs (eg, physical activity or nutritional habits), without a psychological component (eg, the study by Carolan-Olah and Sayakhot [17]).
5. They conducted only cognitive trainings or tasks focused on executive functions, such as cognitive remediation therapy (CRT; eg, the study by Brockmeyer et al [18]). As CRT is fundamentally distinct in nature from psychological and psychoeducational interventions, adding CRT would have made it challenging to compare the treatments meaningfully. However, we included studies with cognitive or executive tasks whenever these were only a part of the intervention, along with psychotherapy sessions (eg, the study by Lansing et al [19]).
6. They were reviews, meta-analyses, protocols, and letters to the editor.

See the figure in the *Results* section for this study’s flowchart.

We used Rayyan software to identify and exclude duplicates among the 3 databases.

From each included article, GG, CG, and SR independently extracted the main characteristics: study author, study title, study design, diagnosis, targeted population, medication, sample size, gender, age, intervention, duration of intervention, follow-up duration, and main results (Table S1 in Multimedia Appendix 2). GG, CG, and SR independently evaluated the strength of reporting with the Quality Assessment Tool for Before-After (Pre-Post) Studies with No Control Group, the Quality Assessment Tool for Case Series Studies, and the Quality Assessment of Controlled Intervention Studies [20].

### Quality of Reporting

The strength of reporting was assessed with the Quality Assessment Tools developed by the National Heart, Lung, and Blood Institute tools [20]. The questions in these tools are designed to help the reviewer focus on the key concepts for assessing the internal validity of a study and concern the objective of this study, the population studied, how it was recruited and measured, the response rate, and the statistical analyses conducted (Tables S2-S4 in Multimedia Appendix 2 for details).

Quality ratings (“good,” “fair,” or “poor”) were assigned based on the following calculation:

- Quality assessment of controlled intervention studies: 0-5 yeses: poor, 6-10 yeses: fair, and 11-14 yeses: good (Table S2 in Multimedia Appendix 2);
- Quality assessment tool for before-after (pre-post) studies with no control group: 0-4 yeses: poor, 5-8 yeses: fair, and 9-12 yeses: good (Table S3 in Multimedia Appendix 2); and
- Quality assessment tool for case series studies: 0-3 yeses: poor, 4-6 yeses: fair, and 7-9 yeses: good (Table S4 in Multimedia Appendix 2).

## Results

### General Description

**Figure 1:**
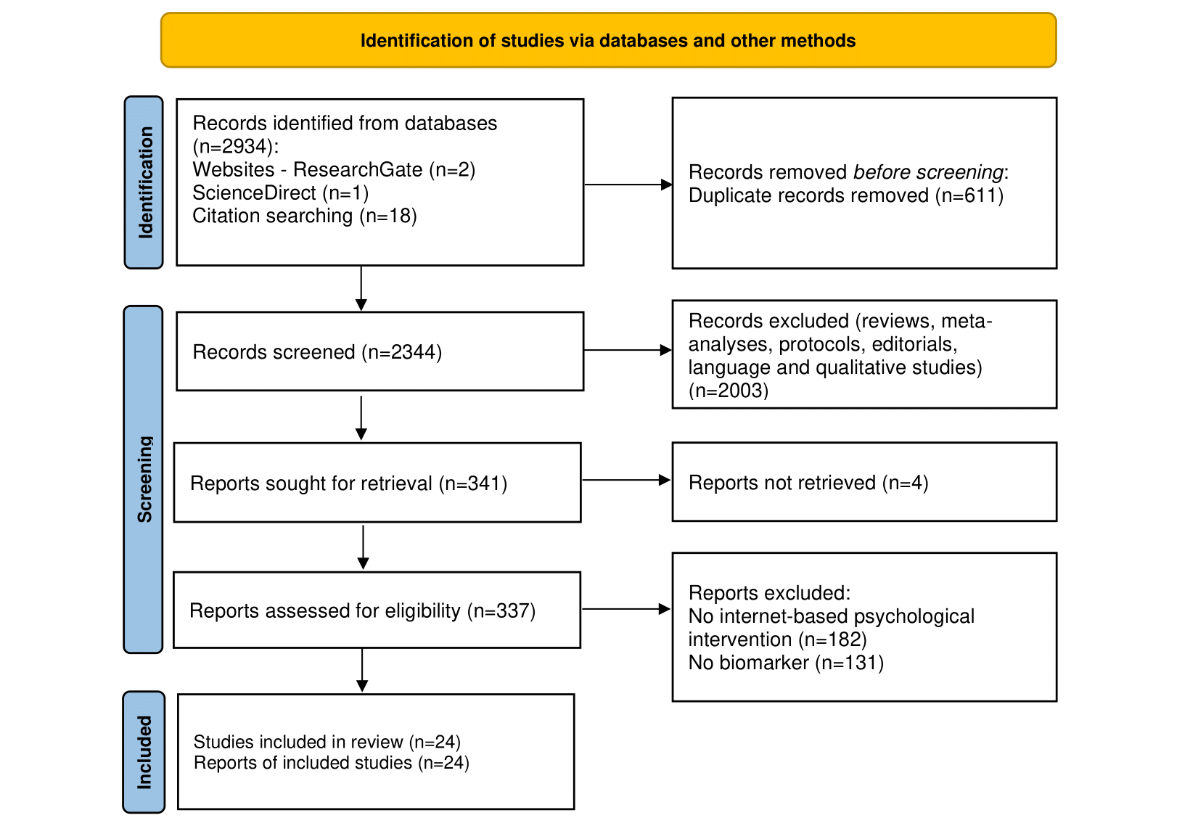
PRISMA (Preferred Reporting Items for Systematic Reviews and Meta-Analysis) study flowchart.

After screening for titles, abstracts, and full texts, we eventually included 24 papers of the 2934 studies identified through the databases and the 21 studies identified through other sources (Figure 1).

The retained studies were either controlled trial intervention studies or randomized controlled trials (n=19, 79%) [21-39], pre-post studies with no control group (n=4, 17%) [19,40-42], or case studies (n=1, 4%) [43].

The interventions or programs were addressed to individuals with a psychiatric or psychological problem (n=6, 25%), individuals with an organic or medical disease (n=10, 42%), and nonclinical populations (n=8, 33%).

The psychiatric or psychological problems included depression (n=3, 13%) [21,22,41], posttraumatic stress disorder (n=2, 8%) [23,24], and sleep problems (n=1, 4%) [42].

The organic or medical diseases included type 1 diabetes (n=3, 13%) [19,25,43], inflammatory bowel disease (n=2, 8%) [26,27], hypertension (n=2, 8%) [28,29], prostate cancer (n=1, 4%) [30], preterm labor (n=1, 4%) [31], and heart diseases (n=1, 4%) [32].

The nonclinical populations included health care professionals (n=3, 13%) [33,34,40], smokers (n=2, 8%) [35,36], university students (n=2, 8%) [37,38], and healthy adult men (n=1, 4%) [39].

Two studies included patients with both a psychiatric or psychological problem and an organic or medical disease [21,42], and in this case, we included the studies under psychiatric or psychological problems.

The psychotherapeutic interventions or programs were CBT or CBT-informed interventions (we included in this category different types of CBTs, such as trauma-focused CBT, CBT for insomnia, behavioral activation treatment, and motivational enhancement therapy CBT, as well as programs and e-counseling based on CBT techniques; n=14, 59%) [19,21-24,28-31,36,39,41-43], mindfulness-based treatments (n=6, 25%) [26,32,34,35,37,40], stress management interventions (n=2, 8%) [27,33], coping skills training (n=1, 4%) [25], and music therapy (n=1, 4%) [38].

The most frequently measured biomarkers were salivary cortisol (sCort; n=7, 29%) [24,30,34,37,39-41], tumor necrosis factor-α (n=5, 21%) [27,30,34,39,42], α amylase (AA; n=4, 17%) [24,39-41], hair cortisol concentrations (HCC; n=4, 17%) [26,32,38,41], interleukin-6 (n=4, 17%) [27,30,34,42], glycated hemoglobin (HbA_1c_) blood glucose level (n=4, 17%) [19,21,25,43], and C-reactive protein (CRP; n=4, 17%) [26,30,34,39]. We grouped the biomarkers measured on their physiological function or meaning, namely stress-related biomarkers, immune response biomarkers, cardiac or cardiovascular biomarkers, electrophysiological biomarkers, inflammatory biomarkers, electrolytes, renal function biomarkers, neurological function biomarkers, and others. Table S5 in Multimedia Appendix 2 provides more details.

Results of the included studies are hereunder presented, divided by the underlying health condition of the targeted population (psychiatric or psychological illnesses, organic or medical diseases, and nonclinical samples), with a primary focus on biomarker variations between groups or in one group after intervention or program.

### Interventions Targeting Psychiatric, Psychological Conditions, or Issues

Two studies found significant improvements in patients with depression after CBT and CBT-informed interventions, showing both reduced chemokines [22] and a decrease in HbA_1c_ blood glucose levels in older adults with type 2 diabetes [21]. In Romero-Sanchiz et al [22], all chemokines decreased in concentration at the study’s end point; however, these differences were observed within participants with mild and moderate depression (mostly those who were medication-free) rather than between patients with severe depression and healthy controls [22]. Concurrently to immune response biomarkers variations, depression scores significantly improved after the internet CBT intervention, showing a relation between chemokine concentrations and depressive symptoms. In Egede et al [21], participants in the telehealth behavioral activation treatment arm showed stable levels of HbA_1c_, as opposed to it increasing in the same-room arm [21]. Moreover, both the telehealth and the same-room treatments equally contributed to lower depression symptoms, with no significant differences between the two study arms.

CBT for insomnia led to significantly increased functional connectivity in bilateral posterior cingulate cortices (PCCs) and right premotor or dorsolateral prefrontal cortex in patients with insomnia [42]. Changes in cytokines levels post interventions were not significant. Participants also reported significant improvements in several sleep quality indices, which correlated with biomarker variations.

The remaining studies providing a CBT intervention to psychiatric samples, targeting patients with depression [41] and posttraumatic stress disorder [23,24], showed no significant changes in biomarkers, particularly stress-related biomarkers [24,41] and oxytocin and vasopressin concentrations [23].

### Interventions Targeting Organic, Medical Conditions, or Issues

Blood pressure (BP) significantly decreased after CBT-informed e-counseling in patients with hypertension [28,29] and in patients with heart disease after a mindfulness-based intervention [32]. Specifically, in Liu et al [28], BP decreased equally in both the e-counseling group and the control group; at 12 months, the e-counseling group, compared to controls, showed a greater reduction in systolic BP (SBP), along with a significant reduction of urinary sodium in females. Similarly, Nolan et al [29] found a significant reduction of BP in both the e-counseling group and the control group, with the variation of SBP being significantly greater in the e-counseling group, compared to controls, at 12 months. Pulse pressure also lessened after 4 and 12 months, while non–high-density lipoprotein cholesterol lowered at 4 months in the e-counseling group. In Gotink et al [32], in patients with heart disease, heart rate (HR), BP, and HCC levels decreased over time, but these changes showed no significant difference between groups, although SBP reduction in the mindfulness group had a greater effect size in the mindfulness group compared to usual care.

HbA_1c_ blood glucose level significantly decreased in patients with type 1 diabetes in 2 studies after CBT interventions [19,43]. However, Kern et al [43] was a case series study on 2 patients and Lansing et al [19] had a sample of 15 patients only. According to Lansing et al [19], a motivational enhancement CBT-informed intervention would benefit glycemic control and HbA_1c_ in teenagers with diabetes [19]. Following the active treatment phase, the HbA_1c_ was indeed reduced compared to its pretreatment levels, whereas during the subsequent maintenance phase, no additional enhancements were observed. Nevertheless, the progress was preserved in most participants. On the contrary, HbA_1c_ slightly increased in youth with type 1 diabetes in one study [25], both in the coping skills training group and in the control one (with no difference between the two), but this variation was only borderline significant (*P*=.05). Although this does not reflect an improvement in metabolic control, the fact that HbA_1c_ did not notably rise in teenagers with diabetes was considered a positive outcome by the authors.

Indoleamine 2,3-dioxygenase significantly decreased in patients with inflammatory bowel disease after a stress management intervention [27]. However, the authors failed to find any change in the many other biomarkers investigated, including brain-derived neurotrophic factor and CRP.

Furthermore, reduced inflammation biomarkers were found in patients with inflammatory bowel disease after mindfulness-based interventions [26]. Participants who received both the blended mindfulness-based intervention and the standard medical therapy presented a significant decrease in fecal calprotectin and CRP compared to participants who received standard medical therapy alone, but there was no significant difference in HCC emerged between the two groups. Immune response biomarkers significantly decreased in men with advanced prostate cancer after 6 months, both in the cognitive behavioral stress management intervention and the health promotion condition, with no significant difference between groups [30]. However, this improvement faced a rebound increase at 6 months. Furthermore, contrary to the authors’ hypothesis, diurnal cortisol diminished only for participants in the health promotion condition after 6 months, while it was higher in men in the cognitive behavioral stress management group. Cortisol levels remained relatively stable over the subsequent 6 months.

Lastly, no significant difference concerning the reduction of sCort levels was found between pregnant women with preterm labor assigned to either an internet-based cognitive behavioral stress management program for preterm labor or a control group based on distraction [31].

### Programs Targeting Nonclinical Populations

A reduction in certain stress-related biomarkers was found after mindfulness [37], but not after music therapy [38]. Specifically, Beerse et al [37] observed a significant reduction in sCort in both the mindfulness-based art therapy group and the neutral clay task control group, as well as a significant decrease in anxiety and perceived stress only in the experimental group. However, no significant correlations between sCort and perceived stress or between sCort and anxiety were detected. Finnerty et al [38] revealed a slight—yet nonsignificant—decrease in the participants’ HCC levels after music therapy, compared to a significant increase in the control group, inferring that the program may enhance stress management skills rather than soothing anxiety [38]. On this behalf, stress and anxiety scores did not change after the 6 weeks program across all kinds of programs and the control group.

Two studies reported heterogeneous changes in immune biomarkers [39,40]. In Heckenberg et al [40], secretory immunoglobulin A increased after a mindfulness-based stress reduction program on direct-care workers, suggesting an improved level of mucosal immunity. The authors also found that changes in salivary AA concentration only approached significance [40].

Schakel et al [39] measured several immune and psychophysiological outcomes in participants who underwent an internet CBT program. After treatment, authors reported a significant increase in immunoglobulin G, a decrease in interleukin-1β and tumor necrosis factor-α, as well as significant variations in a diverse number of chemokines and cytokines. Moreover, participants reported improvements in HR variables over time, but with no significant differences between conditions. Ultimately, HR variability, sCort, AA, and skin conductance showed neither a significant main effect of time nor an interaction between time and condition [39].

Two of the included studies focused on the smoking population and they both reported significant findings [35,36]. In Janes et al [35], posterior cingulate cortex reactivity to smoking cues decreased after both the mindfulness training and the control program, with no significant difference between groups [35]. Participants also reported a decrease in cigarette consumption after the program. On the other hand, in Webb et al [36], 7-day point prevalence abstinence (PPA), defined as self-reported abstinence from smoking for 7 days, displayed higher rates in the program condition compared to the controls at 26 weeks, but this effect did not last up until the 52 weeks’ time point. The abstinence was biochemically verified through breath CO levels, which emerged to be consistent with participants’ self-evaluation, to the advantage of the program condition versus the control group after 26 weeks, but not after 52 weeks. Additionally, the consecutive 7-day PPA, defined as 2 or more consecutive 7-day PPA observations, resulted in significant enhancement in the program arm rather than in the control group both at 26 and 52 weeks.

Lastly, the remaining studies did not report significant changes in biomarkers. Specifically, stress-related biomarkers were found to be substantially the same before and after both a stress management program [33] and mindfulness-based intervention [34].

### Additional Health Outcomes Associated With Biomarker Variations

Although a significant variation in biomarkers can be useful in assessing treatment response, the efficacy of the intervention can be further reinforced if the changes in the biomarkers are associated with a relevant improvement in participants’ health outcomes. Some of the included studies reported, along with a significant variation in biomarkers, a reduction of patients’ symptoms (in the clinical samples) or an overall improvement of participants’ well-being (in the nonclinical samples), reflecting a fairly solid response to the intervention. Hereafter, we summarize the main outcomes of the above-mentioned studies.

Regarding the psychiatric or psychological samples, in Romero-Sanchiz et al [22], the significant decrease in chemokines in the CBT intervention in patients with depression matched a reduction in their symptoms. In Park et al [42], CBT for insomnia successfully improved sleep quality in patients on dialysis with sleeping problems. This achievement correlated with the enhancement, between the pre- and postintervention time points, of the resting-state brain connectivity between the default mode network and the premotor or dorsolateral prefrontal cortex, cortical areas crucially involved in sleep regulation. Among the medical samples, in Liu et al [28], cardiovascular outcomes were associated with improvements in physical activity: specifically, a greater number of daily steps was related to a lower BP. Furthermore, Lansing et al [19], in addition to a decreasing HbA_1c_ after the intervention, reported improvements in teens’ performance on executive functions, specifically working memory and inhibitory control tasks. Fewer errors in inhibitory control tasks significantly correlated with the decreased HbA_1c_ (as well as with the increased frequency of glycemic control). Lastly, regarding nonclinical samples, Janes et al [35] reported a significant correlation between the reduction of PCC reactivity to smoking cues and the decrease in cigarette consumption: although this biomarker decreased in both groups, only in the mindfulness training condition did participants who exhibited a reduction in PCC cue reactivity also showed a greater reduction of cigarette use. Nevertheless, in the clinical samples, the patients’ underlying condition (whether psychological or medical) could cause biological alterations. This makes it difficult to ascertain whether—and to what extent—the significant variations in biomarkers measured in the studies are due to the intervention, rather than the disorder itself.

### Predictors of Response

Regarding the issue of prediction of response, some of the studies investigated whether changes in biomarkers or their baseline values predicted an improvement in symptoms (typically with linear regressions). Janes et al [35] investigated whether variations in PCC reactivity to smoking cues predicted individual-level smoking reduction. Linear regressions were performed, with ΔPCC (the change in PCC reactivity from baseline to post intervention) significantly predicting changes in cigarettes smoked in the mindfulness group. This predictor was not significant in the control group. However, this model was only significant at the individual level, not at the group level. Laufer et al [41] assessed the predictive value of salivary AA, sCort, and hair cortisol before intervention on treatment response to identifying prescriptive biological markers. No correlation was found between changes in biomarkers and changes in depressive symptoms. However, exploratory analysis found that baseline AA predicted treatment response. Nolan et al [29] reported that total lipoprotein cholesterol and SBP were predictors of cardiovascular risk index after the intervention [29]. Finally, Graham et al [34] failed to report associations between changes in perceived stress and changes in CRP and interleukin-6.

### Quality of Reporting

The quality of reporting evaluation is reported in Tables S2-S4 in Multimedia Appendix 2. Four studies were rated as “good” per quality of reporting, 16 studies as “fair,” and 4 studies as “poor.”

Some response patterns that emerged depending on the type of study should be underscored. In the 4 “before-after” studies, 75% (n=3) did not enroll all eligible participants (item 4) and 75% (n=3) did not have a large enough sample size (item 5); additionally, it was not possible to apply a few items to assess: the blindness of people assessing outcomes in 100% (n=4) of the studies (item 8), the percentage of dropout at follow-up in 75% (n=3) of the studies (item 9) and the individual-level outcome efforts in group-level interventions in 100% (n=3) of the studies (item 12). In 19 controlled intervention studies, 74% (n=14) were not double-blind (item 4), 58% (n=11) did not report intervention adherence measures (item 9), 58% (n=11) had a dropout rate higher than 20% (item 7), 47% (n=9) did not have a large enough sample size (item 12), and 47% (n=9) did not use an intention-to-treat (ITT) analysis (item 14).

## Discussion

### Principal Findings

The main goal of this systematic review was to investigate the effects of internet-based psychological interventions on various biomarkers. In addition, we aimed to explore whether variations in such biomarkers reflected alterations of the underlying health condition (whether psychiatric or psychological, organic or medical, or nonclinical). After the screening process, we included 24 studies, which were mainly randomized controlled trials. The most frequently targeted populations were patients who are depressed (for psychological or psychiatric samples), patients who are diabetic (for organic or medical samples), and health care professionals (for nonclinical samples); the most frequently administered programs were internet-based CBT or CBT-informed interventions; and the most frequently measured biomarkers were stress-related biomarkers.

### Effect of Intervention or Program Duration on Biomarker Variations

Internet- and app-based interventions can extensively differ per intervention duration and sessions’ frequency. This produces a variety of conditions that may affect outcomes in different ways. The 24 studies included in this review were rather heterogeneous: except for 4 (17%) studies that provided 12-month interventions or programs [21,28,29,36] and 1 (4%) study with a 25-week-long program [19], in the remaining papers (n=19, 79%) [22-27,30-35,37-43], the treatments lasted no longer than 3 months. Furthermore, among the included studies, the most frequently administered interventions were CBT and mindfulness-based therapy, which are typically short-term treatments.

A brief intervention or program may represent an obstacle to significant findings, considering that some biomarkers might need a longer amount of time to significantly vary, perhaps longer than a brief treatment may allow. All the abovementioned studies that deliver interventions or programs that last 6 to 12 months report significant biomarkers’ changes, as opposed to the 8 (42%) out of 19 treatments [22,26,32,37-40,43] that lasted no longer than 3 months. This may suggest that the longer the intervention or program is, the more likely it is that biomarkers will change.

Nonetheless, we should acknowledge that this does not always occur; in fact, some shorter interventions and programs still report biomarkers’ changes: for example, Park et al [42], Janes et al [35], Beerse et al [37], Schumacher et al [24], and Schakel et al [39], whose treatments lasted 3, 4, 5, 5, and 6 weeks, respectively.

Potential methodological limitations should also be discussed. Follow-up may impact the data collected by researchers in various ways: biological samples (such as saliva or blood samples) might deteriorate with time and become unusable, if not forthwith analyzed; similarly, data might be lost due to misplacement. A further issue is the possible dropout of participants, who may not be reachable or might refuse to partake in the follow-up data collection, leading to a loss of statistical power in the conducted analysis. Therefore, variability of biomarkers at follow-up might stem from different sources, and methodological issues should be considered when interpreting results.

### Effect of Follow-Up on Biomarker Variations

In light of the abovementioned topic, it should also be taken into consideration whether the studies provide a follow-up assessment to evaluate the possibility of a further alteration of biomarkers after a certain amount of time from the intervention end point. Eight (33%) [23-26,30,32,34,39] of the 24 included papers re-evaluated participants at follow-up, yet only 4 (17%) [26,30,32,39] also reported biomarkers’ changes.

Unfortunately, the scarcity of follow-up biomarkers’ evaluations in the included studies and the gaps in measurements in those that provided a follow-up do not allow us to examine in depth the influence of time on the variations of several biological indicators. It is reasonable to think that changes in biomarker levels might depend on a multitude of factors, such as the type of intervention or program, its duration and frequency, and the possible presence of a psychiatric or medical condition, besides the nature of the biomarker itself and the length of follow-up. We need a more homogeneous group of studies to derive more definitive and reliable conclusions.

### Augmentation

According to the reviewed literature, there is growing evidence suggesting that internet-based interventions and programs may be effective in improving a variety of health conditions, even producing significant alterations of some biological factors. Among the several biomarkers included in this systematic review, those that more frequently varied as a consequence of an online treatment or program were cardiac or cardiovascular biomarkers (especially BP and HR), inflammatory biomarkers, neurological function biomarkers, and HbA_1c_. Considering the type of internet-based interventions, CBT or CBT-informed and mindfulness-based interventions proved to be most efficient in improving both biomarkers and symptoms. However, this result might be biased, since these two psychological interventions were also the most represented among the included studies. These effects should not be underestimated in scientific research: when investigating the efficacy or effectiveness of a treatment or program, authors should take into consideration whether participants are currently following an online psychosocial intervention or program, seeing as this might represent a confounding variable. On the same note, internet-based interventions and programs also have implications in clinical practice and could be combined with in-person or pharmacological therapy in the treatment of both psychiatric or psychological and medical conditions [44]. Long-term interventions and programs may be useful to maintain symptom reduction in time and prevent relapses, promoting a more holistic approach to patient care. Furthermore, online or app-based interventions and programs could also be beneficial per prevention, for example, increasing motivation in smokers to quit smoking or teaching health care providers effective strategies to manage stress and anxiety. Given their promising qualities, future research should invest more in studying the long-term effects of internet-based interventions and programs: ideally, including larger and more diverse populations would further enrich our preliminary findings, by extending the generalizability of our results.

### Strengths and Limitations

To the best of our knowledge, this is the first systematic review exploring how biomarkers change because of internet-based psychological interventions and programs. One of the strongest assets of this research regards the fact that it was conducted by following a meticulous and defined methodology according to the PRISMA statement [14]. By the employment of 3 databases and the workforce of 3 independent evaluators, who executed the screening taking into consideration three languages, we managed to reach a satisfying number of studies (n=24). This should be deemed as a major strength, especially in light of internet-based interventions and programs being a relatively new approach.

Nevertheless, conducting this systematic review presented some challenges. The first and main limitation of this study is that the included papers were greatly heterogeneous per targeted populations, administered interventions or programs, and measured biomarkers. Regarding online interventions, we included a variety of psychological, psychosocial, and motivational programs, although the most frequent were CBT or CBT-informed treatments. Such programs also varied per type of material (eg, videos, reading material, messages, etc) and methods of administration (eg, computer, smartphone app, etc). Another source of heterogeneity stems from the vastly different clinical and nonclinical conditions targeted by the interventions. To avoid possible confusion in reporting such variability, we attempted to mitigate this inconvenience by reporting our results separately for psychiatric or psychological, medical, and nonclinical populations: this clustering method allowed us to better describe and appreciate the different effects that various interventions and programs have on the different examined populations. This heterogeneity becomes even more conspicuous when it comes to biomarkers assessment (Table S5 in Multimedia Appendix 2), making it highly difficult to draw comprehensive conclusions. However, we believe that having an entirely homogeneous sample per biomarker (eg, only stress-related biomarkers or only immune response biomarkers) would provide a narrow perspective of the effect of the intervention, for the scope of our review. A second limitation of this review concerns a methodological fault, namely our inability to retrieve information regarding the agreement between authors in case of conflict when screening articles and assessing their suitability for inclusion (Cohen κ).

Other limitations that should be discussed are not problems of this review itself, but they mainly concern inherent weaknesses of the included studies. First, many studies (n=10, 42%) [30,31,33,34,36-38,40,41,43] relied at least on 1 self-measured biomarker assessment, whether it was by collecting saliva samples (eg, the studies by Graham et al [34] and Beerse et al [37]), app and sensor monitoring (eg, the studies by Bauman et al [33] and Finnerty et al [38]), or by accounting for their self-measured biomarkers values (eg, the study by Kern et al [43]). Self-reporting, especially when it comes to biomarkers, is much more likely to affect the reliability of the collected data. This consideration may not apply to app and sensor monitoring, which were deemed as accurate and sensitive methods by the studies that used them (n=3, 13%) [33,36,38], and therefore, we can safely assume that the results were not gravely affected by them. However, it is undeniable that other self-report methods can potentially interfere with the reliability of data, because participants may not be entirely truthful about their reported values or incorrectly execute the sample collection.

Second, another flaw of most of the included studies is the absence of a follow-up evaluation. As previously stated, depending on the underlying condition and the type of outcome measures, some biomarkers may take longer to show a significant alteration. Therefore, some of the studies that did not find significant changes in biomarkers might have shown different results in the longer term. In the wake of this, in studies in which biomarkers vary significantly, a follow-up would allow us to examine how such improvements develop through time. Long-lasting effects of internet-based interventions and programs could help to avoid relapses and delay medical deterioration. Maintenance therapy, if permitted by the theoretical framework of the intervention or program, could be useful to preserve the treatment’s or program’s beneficial effects for a more extended period. Supporting this idea, in Lansing et al [19], the active treatment phase was followed by a maintenance period, which helped sustain the obtained results for 14 additional weeks after treatment ended [19].

Finally, the high dropout rate is another weakness of the included studies that needs to be addressed: as already mentioned in the quality assessment and visible in Tables S2-S4 in Multimedia Appendix 2, as far as it concerns the controlled intervention studies (n=19), more than half of the studies (n=10, 53%) have an overall dropout rate higher than 20%. Higher dropout rates can affect internal validity since they subtract potential additional data. In this review, the high dropout rate may be due to the internet-based nature of the interventions and programs. In face-to-face interventions or programs, the relevance in dropout prevention of a strong therapeutic alliance that encourages compliance has been well documented [45]. Perceiving their therapist as more understanding, involved, and agreeable about their goals may be a protective factor for patients against dropout [46]. Internet-based interventions and programs, especially if self-guided, often lack a relational component, which undoubtedly plays a fundamental role in face-to-face interventions and programs. This may lead to loss of motivation in individuals who find the treatment particularly challenging from an emotional standpoint. Karyotaki et al [3] found that therapist-assisted online interventions or programs have lower dropout rates than self-guided ones. In light of this knowledge, clinicians using internet-based interventions or programs should commit to creating better opportunities to establish a solid alliance with patients, build up strong motivation, and discourage attrition.

### Future Research Directions

Even though our review allowed us to reach some notable findings, this research field is quite recent and deserves further development.

As previously stated, internet-based interventions and programs represent a fairly new answer to those seeking mental health or psychological support for a variety of conditions. The COVID-19 pandemic has ultimately increased this need, by both compelling social distance and worsening pre-existent mental health issues in the general population [47]. This, among many others, is one of the reasons why we managed to include only a moderate number of studies. Due to the continuous development of new technologies and the growth of demand for long-distance services, a great deal of research is expected to come along in the next few years, which will hopefully enrich this field of investigation.

Another research domain that should be further explored, especially in psychological terms, concerns biomarkers. Biological indicators are often used to study organic and medical pathologies but quite rarely when it comes to the psychological and mental health field. A stronger body of scientific research on the biology of mental illnesses may allow us, from a clinical point of view, to reach both a better understanding of their underlying mechanisms and a more suitable way of treating them by adding fundamental new information to the pharmacology area. For these reasons, it is our best hope that future researchers will invest more in using biomarkers as a valid and reliable outcome measure for mental health issues as well.

### Conclusion

Our review provides a synthesis of literature examining the effects of internet-based psychological treatments and programs on several biomarkers. The results suggest that such interventions may have some beneficial effects on a variety of health conditions, in some cases even presenting significant physiological changes. Nonetheless, some studies show little to no positive effects, and in general, the existing evidence is scarce and somewhat conflicting. Although further evidence is required, we hope to raise awareness on the potential impact of internet-based interventions on biomarkers related to mental and physical health.

## Appendix

Multimedia Appendix 1 Search terms.

Multimedia Appendix 2 Supplementary tables S1-S5.

Multimedia Appendix 3 PRISMA (Preferred Reporting Items for Systematic Reviews and Meta-Analyses) checklist.

## Abbreviations

AA: α amylase
BP: blood pressure
CBT: cognitive behavioral therapy
CRP: C-reactive protein
CRT: cognitive remediation therapy
FDA: US Food and Drug Administration
HbA1c: glycated hemoglobin
HCC: hair cortisol concentration
HR: heart rate
ITT: intention-to-treat
NIH: National Institutes of Health
PCC: posterior cingulate cortex
PPA: point prevalence abstinence
PRISMA: Preferred Reporting Items for Systematic Reviews and Meta-Analysis
SBP: systolic blood pressure
sCort: salivary cortisol
