# Supplementary material for: Biomarkers of Response to Internet-Based Psychological Interventions: Systematic Review
Source: J Med Internet Res. 2024 Nov 29;26:e55736. doi: 10.2196/55736 (PMC11645513; doi:10.2196/55736)
Supplement: Multimedia Appendix 1 [file jmir_v26i1e55736_app1.docx]

Search terms

(biomarker* OR biosignature OR “biological marker*” OR “physiological correlate*” OR “cortisol awakening response” OR "physiological measure*" OR "heart rate variability" OR "cortisol stress response" OR imaging OR "galvanic skin response" OR cytokine OR “skin conductance response” OR bioindicator* OR “biological indicator*” OR biomechanism* OR “biological mechanism*” OR “biological predictor*” OR blood OR saliva OR “genetic marker*” OR “inflammatory response” OR “inflammatory biomarker*” OR neurotransmitter* OR “blood-based biomarker*” OR “enzyme activity”) AND (“internet based therap*” OR “internet-based therap*” OR “internet based psychotherap*” OR “internet-based psychotherap*” OR “internet based intervention*” OR “internet-based intervention*” OR “internet-based treatment” OR “internet based treatment” OR “web based therap*” OR “web-based therap*” OR “web based psychotherap*” OR “web-based psychotherap*” OR “web-based treatment” OR “web based intervention*” OR “web based treatment” OR “web-based intervention*” OR “digital therap*” OR “digital psychotherap*” OR “distance counselling” OR “distance psychotherap*” OR “remote therap*” OR “virtual therap*” OR “virtual treatment*” OR “internet-delivered cognitive behavioral therap*” OR “app-based intervention*” OR “app-based therap*” OR “app-based treatment” OR “app based treatment” OR “app based therap*” OR “app based intervention*” OR “computer assisted psychotherap*” OR “computer-assisted psychotherap*” OR “computer-assisted treatment” OR “computer assisted therap*” OR “computer-assisted therap*” OR “computerized therap*” OR “computerized training” OR “computerized treatment” OR “digital mental health intervention*” OR “digital mental health treatment” OR “digital mental health platform*” OR “online intervention*” OR “online psychotherap*” OR “online therap*” OR “online treatment”)
