# Supplementary material for: Biomarkers of Response to Internet-Based Psychological Interventions: Systematic Review
Source: J Med Internet Res. 2024 Nov 29;26:e55736. doi: 10.2196/55736 (PMC11645513; doi:10.2196/55736)
Supplement: Multimedia Appendix 2 [file jmir_v26i1e55736_app2.docx]

Supplementary Tables S1 to S5

This is a Multimedia Appendix to a full manuscript published in the J Med Internet Res. For full copyright and citation information see http://dx.doi.org/10.2196/jmir.xxxx

| **Table S1.** Main features of the studies included. | | | | | | | | | | | |
| --- | --- | --- | --- | --- | --- | --- | --- | --- | --- | --- | --- |
| **Study Author** | **Study Title** | **Diagnosis** | **Targeted population** | **Medication** | **Sample size** | **Gender** | **Mean Age** | **Intervention** | **Duration of intervention** | **Follow up** | **Main**  **results** |
| **RCT/ CONTROLLED TRIALS STUDIES** | | | | | | | | | | | |
| Baumann et al. (2023) | Efficacy of Individualized Sensory-Based mHealth Interventions to Improve Distress Coping in Healthcare Professionals: A Multi-Arm Parallel-Group Randomized Controlled Trial | Non-clinical population | Healthcare professionals | Not Assessed | N=170 | Female: N=104 (41.2%)  Male: N=66 (38.8%) | 42.7±10 years | Experimental Group:  Group 1: Web-based digital stress management intervention  Group 2: Web-based need- oriented digital stress management intervention  Group 3: Web-based need- oriented digital stress management intervention with telephone coaching  Group 4: App-based personality specific digital stress management interventions with sensory biofeedback  Group 5: App-based personality specific digital stress management  intervention with sensory biofeedback and health report  Control Group: participants are assigned to the waitlist and will start the intervention after 8 weeks. | 8 weeks | no | All stress-related HRV parameters did not significantly vary over time, nor differ among study arms. |
| Beerse et al. (2020) | Therapeutic psychological and biological responses to mindfulness-based art therapy | Non-clinical population | full-time students at a public university | Not Assessed | N=77 | Female: N=66 (85.7%)  Male:  N=11  (14.3%) | 19.70±1.56 years | Treatment Group: Mindfulness-  Based Art Therapy (MBAT) intervention, consisting of brief mindfulness practices paired with complementary MBAT art directives using earth-based clay.  Control Group: Neutral Clay Task (NCT), in which participants were instructed to manipulate the clay for 15 minutes. | 5 weeks | no | SCC significantly decreased in both MBAT and control group after intervention, but it’s likely that time, rather than group condition, contributed to such differences. |
| Egede et al. (2018) | Effect of psychotherapy for depression via home telehealth on glycemic control in adults with type 2 diabetes: Subgroup analysis of a randomized clinical trial | Psychiatric-Psychological population | older adults with major depression and type 2 diabetes | Assessed | N=90 | Female: N=2 (2.2%)  Male: N=88 (97.8%) | 63.1±4.2 years | Treatment group: Behavioural Activation Treatment (BAT), based on CBT for depression.  Both groups received the same treatment: one through telemedicine and one at the local Veterans Affairs Medical Center. | 12 months | no | Treatment group had a significant effect on mean A1c, which remained stable in the telemedicine-delivered BAT group, while it increased in the same-room group. Depression scores did not vary significantly over time. |
| Engel et al. (2021) | Associations between oxytocin and vasopressin concentrations, traumatic event exposure and posttraumatic stress disorder symptoms: group comparisons, correlations, and courses during an internet-based cognitive-behavioural treatment | Psychiatric-Psychological population | active and  former male service members of the German Armed Forces | Assessed | N=100 | Male: 100% | 34.76 years | Treatment Group: internet-based therapist-guided trauma-focused cognitive behavioral therapy (TF-CBT).  Control Group: participants were assigned to the waitlist condition and received the TF-CBT intervention after 6 weeks.  Additionally, there were two other control groups who didn’t receive any treatment, consisting of deployed and non-deployed healthy controls. | 5 weeks | 3 months | Mean oxytocin and vasopressin did not differ between groups nor did they change from pre- to post-treatment and follow-up and both were not stable within individuals over time. PTSD symptoms were stable over time and they were not correlated with endocrine parameters. |
| Finnerty et al. (2023) | Online group music therapy:  proactive management of  undergraduate students’ stress  and anxiety | Non-clinical population | full-time undergraduate students, aged 18–24 | Assessed | N=84 | Female: N=69 (82.1%)  Male: N=15 (17.9%) | 20 years | Treatment group: online therapy facilitated by a registered psychotherapist.  Group 1: online Active music therapy  Group 2: online Receptive music therapy  Group 3: online Verbal based therapy (standard of care)  Control Group: two control groups, one assigned to the waitlist and one which received no intervention. | 6 weeks | no | Stress scores did not change after the 6 weeks treatment and were not significantly different between music therapy and control group; cortisol slightly decreased in the therapy groups but it increased significantly in the control group after the intervention. |
| Gonzalez-Moret et al. (2020) | The effect of a mindfulness-based therapy on different biomarkers among patients with inflammatory bowel disease: a randomized controlled trial | Medical population | inflammatory bowel diseases (IBDs);  Crohn’s disease (CD);  ulcerative colitis (UC) | Assessed | N=57 | Female: N=38 (66.7%)  Male: N=19 (33.3%) | 46.2 years | Treatment group: Mindfulness Based Intervention (MBI), consisting of four internet-based therapy modules and four in-person support sessions, administered by a psychologist.  Control group: Standard medical therapy | 8 weeks | 6 months | CRP and FC inflammation biomarkers decreased at 6 months’ follow-up in the MBI group compared to the SMT group.  MBI did not significantly affect the hair cortisol levels at follow-up. |
| Gotink et al. (2017) | Online mindfulness as a promising method to improve exercise capacity in heart disease: 12-month follow-up of a randomized controlled trial | Medical population | Adult patients, between 18 and 65 years of age, with existing diagnosed heart disease (ischemic, valvular, congenital heart disease, or cardiomyopathy) | Not Assessed | N=324; | Female: N=150 (46.3%)  Male: N=174 (53.7%) | 43.2 years | Treatment group: Structured online mindfulness training in which participants were taught different meditations, self-reflection, yoga exercises  Control group: participants received usual care by their treating cardiologist. | 12 weeks | 12 months | In the intention-to-treat analysis, heart rate, systolic and diastolic blood pressure and hair cortisol level decreased over time, but with no significant between-groups effect, as well as anxiety, depression and stress levels. |
| Graham et al. (2022) | Online, low-volume meditation does not alter immune-related biomarkers | Non-clinical population | nurses and nursing assistants | Assessed | N=61 | Female:  N=57 (93.4%)  Male:  N=4  (6.6%) | 33 years | Treatment group: Stress Free Now for Healers, an online stress management program designed to reduce stress by fostering the development of mindfulness  Control group: participants were instructed to quietly listen to relaxing music while doing nothing else for at least 5 min every day for 6 weeks. | 6 weeks | 6 weeks | No biomarker (plasma concentrations, CAR, cortisol slope, cortisol AUC, or dhea: cortisol ratio) significantly changed in nurses in the mindfulness program compared to the control group. Stress levels significantly decreased in the control group after 6 weeks and in the mindfulness group at 12 weeks follow up. |
| Janes et al. (2019) | Quitting starts in the brain: a randomized controlled trial of app-based mindfulness shows decreases in neural responses to smoking cues that predict reductions in smoking | Non-clinical population | individuals who smoke | Assessed | N=67 | Female:  N=45  (67.2%)  Male:  N=22  (32.8%) | 44.5 years | Treatment Group: App-based Mindfulness Training (MT) program, a phone app designed to help users deal with their smoking habits through mindfulness.  Control Group: National Cancer Institute’s QuitGuide app (NCI), a smoking cessation website. | 4 weeks | no | PCC reactivity to smoking cues decreased after both interventions, with no difference between groups. In the MT group, this reduction was correlated with a decline in cigarette consumption, specifically in women. |
| Jaser et al. (2013) | Mediators of 12-month outcomes of two Internet interventions for youth with type 1 diabetes | Medical population | Youth with type 1 diabetes | Not assessed | N=320 | Female: N=177 (55%) Male: N=143 (45%) | 12.3±1 years | Treatment group: TEENCOPE, a coping skills training intervention in a graphic novel format that aims to teach various coping skills.  Control group: Managing Diabetes, an educational intervention. | 5 weeks | 12 months | HbA1c did not significantly change in either the TEENCOPE or the Managing Diabetes intervention. Treatment effects on quality of life were mediated by self-efficacy in both interventions, by stress reactivity, primary and secondary control coping in the coping skills group, and by social acceptances in the diabetes education group. |
| Liu et al. (2020) | Effects of self-guided e-counseling on health behaviors and blood pressure: Results of a randomized trial | Medical population | adults diagnosed with stage 1 or 2 hypertension and between the ages of 35-74 years. | Not Assessed | N=264 | Female: N=154 (58.3%)  Male: N=110 (41.7%) | 57.6 years | Treatment group: e-Counseling intervention based on motivational interviewing and cognitive-behavioral therapy to  promote adherence to self-care behaviors. Control group: participants received each session as an email newsletter article, aiming to improve their self-help skills and lifestyle. | 12 months | no | BP decreased in both the e-Counseling group and the control group. Group differences were only found in SPB after 12 months. Urinary sodium levels showed a significant difference between conditions only in females. At 12-months, daily steps significantly differed from baseline in the eCounseling group. |
| Nolan et al. (2018) | Randomized Controlled Trial of E-Counseling for Hypertension: REACH | Medical population | Stage 1 or 2 hypertension | Assessed | N=264 | Female:  N=154  (58%)  Male:  N=110  (42%) | 57.6 years | Treatment group: e-Counseling intervention based on motivational interviewing and cognitive-behavioral therapy to  promote adherence to self-care behaviors.  Control group: participants received each session as an email newsletter article, aiming to improve self-help skills and lifestyle. | 12 months | no | BP significantly decreased in both groups at 4 and 12 months. Changes of SBP were significantly higher in the e-Counseling group, compared to controls, only at 12 months. Changes in DBP did not significantly differ between groups at any point in time.  PP reduction from baseline was significant for e-counseling and control at 4 and 12 months. Lipoprotein cholesterol did not change significantly in either group.  Significantly lower non–HDL-C and a trend toward significantly lower TC at 4 months was observed for e-counseling versus control. |
| Peerani et al. (2022) | A randomized controlled trial of a multicomponent online stress reduction intervention in inflammatory bowel disease | Medical population | patients with inflammatory bowel disease, with either ulcerative colitis (UC) or Crohn’s disease (CD) | Assessed | N=101 | Female:  N=76 (75%)  Male:  N=25  (25%) | 42.5±14.1 years | Treatment Group: stress reduction intervention with meditation and CBT or positive psychology-based videos.  Control Group: participants received weekly themed emails containing a motivational quote and they were given the option of taking part in the intervention after 12 weeks. | 12 weeks | no | From baseline to study end, only IDO significantly decreased in the intervention group. PSS significantly improved after the stress reduction intervention, and various improvements were observed in mental health, resilience and health-related quality of life. |
| Penedo et al. (2021) | Effects of web-based cognitive behavioral stress management and health promotion interventions on neuroendocrine and inflammatory markers in men with advanced prostate cancer: A randomized controlled trial | Medical population | men with stage III or IV prostate cancer | Not Assessed | N=192 | Male: 100% | 68.84 years | Treatment Group: Cognitive Behavioral Stress Management (CBSM), a group-based intervention that integrates cognitive behavioral stress- and self-management skills with relaxation skills training.  Control Group: Health Promotion (HP) intervention that integrates didactic presentations related to living with advanced prostate cancer. | 10 weeks | 6 months and 12 months | IL-10, IL-8 and TNF-α significantly decreased after 6 months in both the CBSM and the health promotion conditions, but they showed a rebound increase from 6 to 12 months. Diurnal cortisol slope was flatter in men in health promotion, compared to CBSM, at 6 months, but not at 12 months. |
| Romero-Sanchiz et al. (2020) | Variation in chemokines plasma concentrations in primary care depressed patients associated with Internet-based cognitive behavioral therapy | Psychiatric-Psychological population | patients with mild and moderate depression and healthy controls | Assessed | N=126 | Female: N=74 (58.7%) Male: N=52 (41.2%) | 39.42 years | Treatment group: internet-delivered self-help program “Smiling is fun”,  with different CBT-based techniques for coping with mild and moderate depression.  Control group: healthy controls did not receive any intervention. | 10 modules | no | From baseline to post-intervention, all chemokines within treated patients were found significantly decreased.  The main differences were found between patients with mild or moderate depression, rather than between patients with severe depression and controls.  The results were coupled with significantly decreased depression scores within depressed patients. |
| Schakel et al. (2020) | An internet-based psychological intervention with a serious game to improve vitality, psychological and physical condition, and immune function in healthy male adults: Randomized controlled trial | Non-clinical population | healthy men 18-35 | Not Assessed | N=69 | Male:  N=69 (100%) | 22.7 years | Treatment group: internet ICBT intervention consisting of homework assignments, asynchronously provided feedback messages and a serious game that included comparable modules as the guided intervention.  Control group: participants did not receive any training. | 6 weeks | 4 weeks | Several cytokines and chemokines significantly varied both in the intervention and the control group between pre-vaccination and end of the test day (post treatment), but no between-group differences were found. IL-1β and TNF-α significantly increased from baseline to test day in the control group, but not in the intervention condition. IgG antibody levels showed a significant increase between baseline and follow up in the intervention condition. Cortisol,  alpha amylase and skin conductance did not change over time, nor between groups. HR significantly decreased at the 4 weeks follow up in the intervention group, compared to the controls. |
| Schumacher et al. (2021) | Salivary Cortisol and Alpha-Amylase in Posttraumatic Stress  Disorder and Their Potential Role in the Evaluation of Cognitive  Behavioral Treatment Outcomes | Psychiatric-Psychological population | German Armed Forces active and former service members with clinical or subclinical PTSD | Assessed | N=102 | Men:  100% | 37.69±9.49 years | Treatment Group: internet-based therapist-guided Trauma-Focused Cognitive Behavioral Therapy (TF-CBT).  Control Group: participants were assigned to the waitlist condition and received the TF-CBT intervention after 6 weeks.  Additionally, there were two other control groups who didn’t receive any treatment, consisting of deployed and nondeployed healthy controls. | 5 weeks | 3 months | Among participants who completed TF-CBT, along with the stability of PTSD symptoms, no statistically significant changes were found in the sCort or the sAA levels both from pretreatment to post-treatment and from post-  treatment to follow-up. |
| Urech et al: (2017) | Efficacy of an internet-based cognitive behavioral stress management training in women with idiopathic preterm labor: A randomized controlled intervention study | Medical population | pregnant women weeks 18–32 and with diagnosed Preterm Labor | Assessed | N=93 | Female N=93 (100%) | 32.6±3.74 years | Treatment Group: online stress management-guided self-help program (IB-CBSM) for pregnant women with preterm labor.  Control Group: six online  sessions in which women were advised to perform various activities provided on the study website. | 6 weeks | no | The analysis of the cortisol levels (CAR AUCg/AUCi) revealed no significant difference between the two groups or over time, as well as no significant interaction effect of “group x time”. |
| Webb et al. (2022) | Long-Term Effectiveness of a Clinician-Assisted Digital Cognitive Behavioral Therapy Intervention for Smoking Cessation: Secondary Outcomes From a Randomized Controlled Trial | Non-clinical population | adult smokers | Assessed | N=556 | Female: N=239  (45.1%)  Male:  N=291 (54.9%) | 41±12 years | Treatment Group: Quit Genius, a digital clinician-assisted CBT  intervention, consisting of a smartphone app with self-guided CBT content and a quit coach who provided reinforcing feedback.  Control Group: very brief advice (VBA) to stop smoking, an evidence-based intervention designed to facilitate quit attempts, coupled with referral to a cessation service. | 52 weeks | no | Participants in the Quit Genius condition self-reported higher 7-day point prevalence abstinence (PPA) rates at weeks 4 and 26, relative to controls. This effect did not last at 52 weeks.  At 4, 26 and 52  weeks expired CO <5 ppm corresponded with participant self-reported abstinence for the majority (93.8%, 93.6% and 92.4%) of participants, respectively. |
| PRE-POST NO CONTROL GROUP STUDIES | | | | | | | | | | | |
| Heckenberg et al. (2018) | An online mindfulness-based program is effective in improving affect, over-commitment, optimism and mucosal immunity | Non-clinical population | direct-care workers | Assessed | N=22 | Female:  N=19  (85.7%)  Male:  N=3  (14.3%) | 43.2±10.2 years | Online-delivered Mindfulness-Based Stress Reduction (MBSR) program, Including weekly reading and videos as well as formal mindfulness meditation. | 8 weeks | no | Online MBSR improved anxiety and fatigue, decreased over-commitment and increased optimism and mucosal immunity.  sIgA increased after treatment, sAA concentration only approached significance, CAR and sAA-AR did not change. |
| Lansing et al. (2016) | Pilot Study of a Web-Delivered Multicomponent Intervention for Rural Teens with Poorly Controlled Type 1 Diabetes | Medical population | teens age 13–17 with type 1 diabetes, average HbA1c ≥ 64 mmol/mol (8%) for past 6 months, most recent HbA1c ≥ 64 mmol/mol (8%), type 1 diabetes duration >18 months | Not Assessed | N=15 | Female: N=7 (47%)  Male: N=8 (53%) | 15.8 years | Active Treatment (ActiveTX), an 11 weeks program including internet-delivered weekly behavior economic incentives (BEI), brief motivational enhancement and cognitive behavioral therapy (MET/CBT) sessions, and working memory training (WMT).  Maintenance Treatment (MaintTX), a following 14-week  program with fading of BEI and MET/CBT sessions. | 25 weeks | no | HbAc1 significantly decreased after active treatment and such results were maintained after the maintenance phase. Performance on working memory and inhibitory control tasks improved at the end of maintenance treatment. |
| Laufer et al. (2023) | The effect of an internet-based intervention for depression on cortisol and alpha-amylase | Psychiatric-Psychological population | mild to moderate major depressive disorder | Assessed | N=46 | Female:  N=38 (82.6%)  Male:  N=8  (17.4%) | 35±12.1 years | Therapist-guided, asynchronous CBT for mild to moderate Major Depressive Disorder (MDD). | 7 weeks | no | Depressive symptoms and chronic stress significantly decreased after the intervention. CAR changed after the intervention but this result was unrobust. No other sCort, sAA and HCC parameters significantly vary. |
| Park et al. 2020 | Changes in resting-state brain connectivity following computerized cognitive behavioral therapy for insomnia in dialysis patients: A pilot study | Psychiatric-Psychological population | patients undergoing maintenance hemodialysis or peritoneal dialysis who had insomnia | Assessed | N=17 | Female: N=13 (76.5%)  Male: N=4 (23.5%) | 45.8±8.6 years | Computerized cognitive behavioral therapy for insomnia (cCBTi). | 3 weeks | no | Functional connectivity increased between the DMN and premotor/DLPFC after intervention. This correlated with improved sleep quality and the decrease of interleukin-1β. |
| CASE STUDIES | | | | | | | | | | | |
| Kern et al. (2022) | Online Guided Self-help Cognitive Behavioral Therapy With Exposure to Anxiety and Problem Solving in Type 1 Diabetes Mellitus: Case Study | Medical population | Type 1 Diabetes Mellitus | Not Assessed | N=2 | Female: N=1 (50%)  Male: N=1 (50%) | Mr A was in his 40s.  Ms B was in her 30s | iCBT with psychoeducation, problem solving, exposure, cognitive restructuring, assertiveness training and life values coupled with the opportunity to contact a therapist for guidance, support, feedback, and questions. | 8 weeks | no | Both participants reported an improvement in HbA1c. Mr. B also reported a significant reduction of diabetes-specific problems, specifically fear of hypoglycemia. |

| **Table S2.** Quality of reporting according to the quality assessment tool of controlled intervention studies (N=19) (NHLBI, NIH, 2021). | | | | | | | | | | | | | | | |
| --- | --- | --- | --- | --- | --- | --- | --- | --- | --- | --- | --- | --- | --- | --- | --- |
| **Study** | **1. Was the study described as randomized, a randomized trial, a randomized clinical trial, or an RCT?** | **2. Was the method of randomization adequate (i.e., use of randomly generated assignment)?** | **3. Was the treatment allocation concealed (so that assignments could not be predicted)?** | **4. Were study participants and providers blinded to treatment group assignment?** | **5. Were the people assessing the outcomes blinded to the participants' group assignments?** | **6. Were the groups similar at baseline on important characteristics that could affect outcomes (e.g., demographics, risk factors, co-morbid conditions)?** | **7. Was the overall drop-out rate from the study at endpoint 20% or lower of the number allocated to treatment?** | **8. Was the differential drop-out rate (between treatment groups) at endpoint 15 percentage points or lower?** | **9. Was there high adherence to the intervention protocols for each treatment group?** | **10. Were other interventions avoided or similar in the groups (e.g., similar background treatments)?** | **11. Were outcomes assessed using valid and reliable measures, implemented consistently across all study participants?** | **12. Did the authors report that the sample size was sufficiently large to be able to detect a difference in the main outcome between groups with at least 80% power?** | **13. Were outcomes reported or subgroups analyzed prespecified (i.e., identified before analyses were conducted)?** | **14. Were all randomized participants analyzed in the group to which they were originally assigned, i.e., did they use an intention-to-treat analysis?** | **Rating** |
| **Baumann et al. (2023)** | **yes** | **yes** | **yes** | **no** | **yes** | **yes** | **no** | **no** | **no** | **yes** | **yes** | **no** | **yes** | **no** | **FAIR** |
| **Beerse et al. (2020)** | **yes** | **nr** | **nr** | **no** | **nr** | **no** | **no** | **yes** | **nr** | **yes** | **yes** | **nr** | **yes** | **no** | **POOR** |
| **Egede et al. (2018)** | **yes** | **yes** | **yes** | **no** | **nr** | **yes** | **nr** | **nr** | **nr** | **yes** | **yes** | **no** | **no** | **no** | **FAIR** |
| **Engel et al. (2021)** | **yes** | **yes** | **nr** | **no** | **no** | **no** | **no** | **no** | **nr** | **yes** | **yes** | **yes** | **yes** | **no** | **FAIR** |
| **Finnerty et al. (2023)** | **yes** | **nr** | **nr** | **no** | **nr** | **nr** | **no** | **nr** | **nr** | **yes** | **no** | **no** | **yes** | **no** | **POOR** |
| **Gonzalez-Moret et al. (2020)** | **yes** | **yes** | **yes** | **no** | **yes** | **no** | **yes** | **yes** | **nr** | **yes** | **yes** | **yes** | **yes** | **yes** | **GOOD** |
| **Gotink et al. (2017)** | **yes** | **yes** | **yes** | **no** | **yes** | **yes** | **no** | **no** | **no** | **yes** | **yes** | **yes** | **yes** | **yes** | **FAIR** |
| **Graham et al. (2022)** | **yes** | **yes** | **yes** | **no** | **no** | **yes** | **yes** | **yes** | **no** | **yes** | **yes** | **yes** | **no** | **no** | **FAIR** |
| **Janes et al. (2019)** | **yes** | **yes** | **yes** | **yes** | **yes** | **yes** | **yes** | **yes** | **nr** | **yes** | **yes** | **yes** | **yes** | **no** | **GOOD** |
| **Jaser et al. (2013)** | **yes** | **nr** | **nr** | **no** | **no** | **yes** | **no** | **yes** | **nr** | **yes** | **yes** | **nr** | **no** | **yes** | **FAIR** |
| **Liu et al. (2020)** | **yes** | **nr** | **nr** | **yes** | **nr** | **yes** | **no** | **yes** | **nr** | **yes** | **yes** | **no** | **yes** | **yes** | **FAIR** |
| **Nolan et al. (2018)** | **yes** | **yes** | **yes** | **yes** | **yes** | **yes** | **no** | **yes** | **nr** | **yes** | **yes** | **no** | **no** | **yes** | **FAIR** |
| **Peerani et al. (2022)** | **yes** | **yes** | **yes** | **no** | **nr** | **no** | **yes** | **yes** | **yes** | **yes** | **yes** | **yes** | **yes** | **yes** | **GOOD** |
| **Penedo et al. (2021)** | **yes** | **nr** | **nr** | **no** | **nr** | **yes** | **yes** | **yes** | **no** | **yes** | **yes** | **no** | **yes** | **yes** | **FAIR** |
| **Romero-Sanchiz et al. (2020)** | **no** | **na** | **no** | **no** | **nr** | **no** | **no** | **na** | **nr** | **no** | **yes** | **no** | **yes** | **na** | **POOR** |
| **Schakel et al. (2020)** | **yes** | **yes** | **yes** | **yes** | **no** | **yes** | **yes** | **yes** | **nr** | **yes** | **yes** | **yes** | **yes** | **nr** | **GOOD** |
| **Schumacher et al. (2021)** | **yes** | **nr** | **nr** | **no** | **no** | **no** | **no** | **no** | **no** | **yes** | **yes** | **no** | **yes** | **no** | **POOR** |
| **Urech et al. (2017)** | **yes** | **yes** | **yes** | **nr** | **nr** | **yes** | **no** | **yes** | **yes** | **yes** | **yes** | **no** | **yes** | **no** | **FAIR** |
| **Webb et al. (2022)** | **yes** | **yes** | **yes** | **no** | **yes** | **yes** | **yes** | **yes** | **no** | **yes** | **no** | **yes** | **no** | **yes** | **FAIR** |

| **Table S3.** Quality of reporting according to the quality assessment tool before-after (pre-post) studies with no control group **(N=5) (NHLBI, NIH, 2021).** | | | | | | | | | | | | | |
| --- | --- | --- | --- | --- | --- | --- | --- | --- | --- | --- | --- | --- | --- |
| **Study** | **1. Was the study question or objective clearly stated?** | **2. Were eligibility/selection criteria for the study population prespecified and clearly described?** | **3. Were the participants in the study representative of those who would be eligible for the test/service/intervention in the general or clinical population of interest?** | **4. Were all eligible participants that met the prespecified entry criteria enrolled?** | **5. Was the sample size sufficiently large to provide confidence in the findings?** | **6. Was the test/service/intervention clearly described and delivered consistently across the study population?** | **7. Were the outcome measures prespecified, clearly defined, valid, reliable, and assessed consistently across all study participants?** | **8. Were the people assessing the outcomes blinded to the participants' exposures/interventions?** | **9. Was the loss to follow-up after baseline 20% or less? Were those lost to follow-up accounted for in the analysis?** | **10. Did the statistical methods examine changes in outcome measures from before to after the intervention? Were statistical tests done that provided p values for the pre-to-post changes?** | **11. Were outcome measures of interest taken multiple times before the intervention and multiple times after the intervention (i.e., did they use an interrupted time-series design)?** | **12. If the intervention was conducted at a group level (e.g., a whole hospital, a community, etc.) did the statistical analysis take into account the use of individual-level data to determine effects at the group level?** | **Rating** |
| **Heckenberg et al. (2018)** | **yes** | **yes** | **yes** | **yes** | **no** | **yes** | **yes** | **na** | **na** | **yes** | **yes** | **na** | **FAIR** |
| **Lansing et al. (2016)** | **yes** | **yes** | **no** | **no** | **no** | **yes** | **yes** | **na** | **yes** | **yes** | **nr** | **na** | **FAIR** |
| **Laufer et al. (2023)** | **yes** | **yes** | **yes** | **no** | **yes** | **yes** | **yes** | **na** | **na** | **yes** | **yes** | **na** | **FAIR** |
| **Park et al. (2020)** | **yes** | **yes** | **yes** | **no** | **no** | **yes** | **yes** | **na** | **na** | **yes** | **no** | **na** | **FAIR** |

| **Table S4.** Quality of reporting according to the quality assessment tool for case series studies (N=1) (NHLBI, NIH, 2021). | | | | | | | | | | |
| --- | --- | --- | --- | --- | --- | --- | --- | --- | --- | --- |
| **Study** | **1. Was the study question or objective clearly stated?** | **2. Was the study population clearly and fully described, including a case definition?** | **3. Were the cases consecutive?** | **4. Were the subjects comparable?** | **5. Was the intervention clearly described?** | **6. Were the outcome measures clearly defined, valid, reliable, and implemented consistently across all study participants?** | **7. Was the length of follow-up adequate?** | **8. Were the statistical methods well-described?** | **9. Were the results well-described?** | **Rating** |
| **Kern et al. (2022)** | **yes** | **yes** | **yes** | **no** | **yes** | **no** | **na** | **no** | **yes** | **FAIR** |

| **Table S5.** List of biomarkers considered in the included studies.  ^a^Post intervention, the biomarker significantly varied:  ^b^ from baseline in one group, and between groups (RCTs)  ^c^ from baseline in all the groups, with no significant difference between them (RCTs)  ^d^ from baseline (pre-post no control group/case study)  ^e^ the variation was in the control group  ^f^ variations were post intervention, between vaccination day and test day  ^g^ only in females | | |
| --- | --- | --- |
| **Studied biomarker** | **N° papers studying it (%)** | **N° papers where result was significant^a^ (%)** |
| STRESS BIOMARKERS | | |
| Salivary Cortisol (sCort) | 7 (29.17%) (Beerse et al., 2020; Graham et al., 2022; Heckenberg et al., 2018; Laufer et al., 2023; Penedo et al., 2021; Schumacher et al., 2021; Schakel et al., 2020) | 1 (4.17%) (Beerse et al., 2020^c^) |
| Hair cortisol concentrations (HCC) | 4 (16.67%) (Finnerty et al., 2023; Gonzalez-Moret et al., 2020; Gotink et al., 2017; Laufer et al., 2023) | 1 (4.17%) (Gotink et al., 2017^c^) |
| Alpha amylase (AA) | 4 (16.67%) (Heckenberg et al., 2018; Laufer et al., 2023; Schumacher et al., 2021; Schakel et al., 2020) | 1 (4.17%) (Heckenberg et al., 2018^d^) |
| Cortisol/alpha-amylase awakening response (CAR) | 3 (12.5%) (Laufer et al., 2023; Schumacher et al., 2021; Urech et al., 2017) | 0 |
| Diurnal cortisol | 1 (4.17%) (Penedo et al., 2021) | 1 (4.17%) (Penedo et al., 2021^b; e^) |
| IMMUNE RESPONSE BIOMARKERS | | |
| Tumor necrosis factor-alpha (TNFα) | 5 (20.83%) (Graham et al., 2022; Park et al., 2020; Peerani et al., 2022; Penedo et al., 2021; Schakel et al., 2020) | 2 (8.33%) (Penedo et al., 2021^c^; Schakel et al., 2020^b; e^) |
| Interleukin (IL)-6 | 4 (16.67%) (Graham et al., 2022; Park et al., 2020; Peerani et al., 2022; Penedo et al., 2021) | 0 |
| Interleukin (IL)-10 | 3 (12.5%) (Graham et al., 2022; Peerani et al., 2022; Penedo et al., 2021) | 1 (4.17%) (Penedo et al., 2021^c^) |
| Interleukin (IL)-8 | 3 (12.5%) (Graham et al., 2022; Penedo et al., 2021; Schakel et al., 2020) | 1 (4.17%) (Penedo et al., 2021^c^) |
| Interferon (IFN)-γ | 1 (4.17%) (Graham et al., 2022) | 0 |
| Interleukin (IL)-1β | 2 (8.33%) (Park et al., 2020; Schakel et al., 2020) | 1 (4.17%) (Schakel et al., 2020^b; e^) |
| Chemokines and cytokines (IL-2, IL-4, IL-16, IP-10, CCL1, CCL2, CCL3, CCL7, CCL8, CCL11, CCL13, CCL15, CCL17, CCL19, CCL20, CCL21, CCL22, CCL23, CCL24, CCL25, CCL26, CCL27, CXCL1, CXCL2, CXCL5, CXCL6, CXCL9, CCL12, CXCL13, CXCL16, CX3CL1, GM-CSF, MIF) | 1 (4.17%) (Schakel et al., 2020) | 1 (4.17%) (Schakel et al., 2020^c; f^) |
| Chemokines plasma concentrations of CXCL12, CCL11, CX3CL1 and CCL2 | 1 (4.17%) (Romero-Sanchiz et al., 2020) | 1 (4.17%) (CXCL12, CCL2) (Romero-Sanchiz et al., 2020^b^) |
| Immunoglobulin g (IgG) | 1 (4.17%) (Schakel et al., 2020) | 1 (4.17%) (Schakel et al., 2020^b^) |
| Secretory immunoglobulin A (sIgA) | 1 (4.17%) (Heckenberg et al., 2018) | 1 (4.17%) (Heckenberg et al., 2018^d^) |
| CTRA gene expression | 1 (4.17%) (Graham et al., 2022) | 0 |
| CARDIAC / CARDIOVASCULAR BIOMARKERS | | |
| Systolic blood pressure (SBP) | 3 (12.5%) (Gotink et al., 2017; Liu et al., 2020; Nolan et al., 2018) | 3 (12.5%) (Gotink et al., 2017^c^; Liu et al., 2020^b^; Nolan et al., 2018^b^) |
| Diastolic blood pressure (DBP) | 3 (12.5%) (Gotink et al., 2017; Liu et al., 2020; Nolan et al., 2018) | 3 (12.5%) (Gotink et al., 2017^c^; Liu et al., 2020^c^; Nolan et al., 2018^c^) |
| Heart rate (HR) | 2 (8.33%) (Gotink et al., 2017; Schakel et al., 2020) | 2 (8.33%) (Gotink et al., 2017^c^; Schakel et al., 2020^c^) |
| Heart rate variability (HRV) | 2 (8.33%) (Finnerty et al., 2023; Schakel et al., 2020) | 0 |
| Pulse pressure | 1 (4.1%) (Nolan et al., 2018) | 1 (4.17%) (Nolan et al., 2018^c^) |
| Non– high-density lipoprotein cholesterol (non–HDL-C) | 1 (4.17%) (Nolan et al., 2018) | 1 (4.17%) (Nolan et al., 2018^b^) |
| Total lipo- protein cholesterol (TC) | 1 (4.17%) (Nolan et al., 2018) | 0 |
| High sensitivity C-reactive protein (hs-CRP) | 1 (4.17%) (Peerani et al., 2022) | 0 |
| N-terminal pro-brain natriuretic peptide | 1 (4.17%) (Gotink et al., 2017) | 0 |
| Low-density lipoprotein cholesterol | 1 (4.17%) (Nolan et al., 2018) | 0 |
| TC/HDL-C ratio | 1 (4.17%) (Nolan et al., 2018) | 0 |
| ELECTROPHYSIOLOGICAL BIOMARKERS | | |
| Skin conductance | 1 (4.17%) (Schakel et al., 2020) | 0 |
| Stress-related heart rate variability parameters:  SDNN;  RMSSD;  LF/HF ratio;  Baevsky Index | 1 (4.17%) (Baumann et al., 2023) | 0 |
| INFLAMMATORY BIOMARKERS | | |
| C-reactive protein (CRP) | 4 (16.67%) (Gonzalez-Moret et al., 2020; Graham et al., 2022; Penedo et al., 2021; Schakel et al., 2020) | 1 (4.17%) (Gonzalez-Moret et al., 2020^b^) |
| Faecal calprotectin (FC) | 1 (4.17%) (Gonzalez-Moret et al., 2020) | 1 (4.17%) (Gonzalez-Moret et al., 2020^b^) |
| Indoleamine 2,3-dioxygenase (IDO) | 1 (4.17%) (Peerani et al., 2022) | 1 (4.17%) (Peerani et al., 2022^b^) |
| ELECTROLYTES | | |
| Urinary sodium | 1 (4.17%) (Liu et al., 2020) | 1 (4.17%) (Liu et al., 2020^b; g^) |
| Potassium | 1 (4.17%) (Park et al., 2020) | 0 |
| Calcium | 1 (4.17%) (Park et al., 2020) | 0 |
| Phosphorus | 1 (4.17%) (Park et al., 2020) | 0 |
| RENAL FUNCTION BIOMARKERS | | |
| Blood Urea Nitrogen (BUN) | 1 (4.17%) (Park et al., 2020) | 0 |
| Creatinine | 1 (4.17%) (Park et al., 2020) | 0 |
| Albumin | 1 (4.17%) (Park et al., 2020) | 0 |
| NEUROLOGICAL FUNCTION BIOMARKERS | | |
| Posterior cingulate cortex (PCC) reactivity | 1 (4.17%) (Janes et al., 2019) | 1 (4.17%) (Janes et al., 2019^c^) |
| Bilateral posterior cingulate cortex (PCCs) and right premotor/dorsolateral prefrontal cortex (DLPFC) | 1 (4.17%) (Park et al., 2020) | 1 (4.17%) (Park et al., 2020^d^) |
| Triggering receptor expressed on myeloid cells 2 (TREM-2) | 1 (4.17%) (Peerani et al., 2022) | 0 |
| Brain-derived neurotrophic factor (BDNF) | 1 (4.17%) (Peerani et al., 2022) | 0 |
| OTHER | | |
| HbA1c (glycated hemoglobin) | 4 (16.67%) (Kern et al., 2022; Jaser et al., 2013; Egede et al., 2017; Lansing et al., 2016) | 3 (12.5%) (Kern et al., 2022^d^; Egede et al., 2017^b; e^; Lansing et al., 2016^d^) |
| Carbon monoxide (CO) levels | 1 (4.17%) (Webb et al., 2022) | 1 (4.17%) (Webb et al., 2022^c^) |
| Hemoglobin | 1 (4.17%) (Park et al., 2020) | 0 |
| Endogenous oxytocin and vasopressin concentrations | 1 (4.17%) (Engel et al., 2021) | 0 |

**References**

1. Baumann H, Heuel L, Bischoff LL, Wollesen B. Efficacy of individualized sensory-based mHealth interventions to improve distress coping in healthcare professionals: a multi-arm parallel-group randomized controlled trial. Sensors (Basel). 2023;23(4):2322. [FREE Full text] [doi: 10.3390/s23042322] [Medline: 36850920]
2. Beerse ME, van Lith T, Stanwood G. Therapeutic psychological and biological responses to mindfulness-based art therapy. Stress Health. Oct 2020;36(4):419-432. [doi: 10.1002/smi.2937] [Medline: 32073202]
3. Egede LE, Walker RJ, Payne EH, Knapp RG, Acierno R, Frueh BC. Effect of psychotherapy for depression via home telehealth on glycemic control in adults with type 2 diabetes: subgroup analysis of a randomized clinical trial. J Telemed Telecare. 2018;24(9):596-602. [doi: 10.1177/1357633X17730419] [Medline: 28945160]
4. Engel S, Schumacher S, Niemeyer H, Kuester A, Burchert S, Klusmann H, et al. Associations between oxytocin and vasopressin concentrations, traumatic event exposure and posttraumatic stress disorder symptoms: group comparisons, correlations, and courses during an internet-based cognitive-behavioural treatment. Eur J Psychotraumatol. 2021;12(1):1886499. [doi: 10.1080/20008198.2021.1886499] [Medline: 33968321]
5. Finnerty R, McWeeny S, Trainor L. Online group music therapy: proactive management of undergraduate students' stress and anxiety. Front Psychiatry. 2023;14:1183311. [FREE Full text] [doi: 10.3389/fpsyt.2023.1183311] [Medline: 37151974]
6. González-Moret R, Cebolla A, Cortés X, Baños RM, Navarrete J, de la Rubia JE, et al. The effect of a mindfulness-based therapy on different biomarkers among patients with inflammatory bowel disease: a randomised controlled trial. Sci Rep. 2020;10(1):6071. [doi: 10.1038/s41598-020-63168-4] [Medline: 32269278]
7. Gotink RA, Younge JO, Wery MF, Utens EMWJ, Michels M, Rizopoulos D, et al. Online mindfulness as a promising method to improve exercise capacity in heart disease: 12-month follow-up of a randomized controlled trial. PLoS One. 2017;12(5):e0175923. [doi: 10.1371/journal.pone.0175923] [Medline: 28486559]
8. Graham B, Jin Y, Bazeley P, Husni E, Calabrese LH. Online, low-volume meditation does not alter immune-related biomarkers. Brain Behav Immun Health. Dec 2022;26:100531. [FREE Full text] [doi: 10.1016/j.bbih.2022.100531] [Medline: 36267832]
9. Heckenberg RA, Hale MW, Kent S, Wright BJ. An online mindfulness-based program is effective in improving affect, over-commitment, optimism and mucosal immunity. Physiol Behav. Feb 01, 2019;199:20-27. [doi: 10.1016/j.physbeh.2018.11.001] [Medline: 30395806]
10. Janes AC, Datko M, Roy A, Barton B, Druker S, Neal C, et al. Quitting starts in the brain: a randomized controlled trial of app-based mindfulness shows decreases in neural responses to smoking cues that predict reductions in smoking. Neuropsychopharmacology. Aug 2019;44(9):1631-1638. [FREE Full text] [doi: 10.1038/s41386-019-0403-y] [Medline: 31039580]
11. Jaser SS, Whittemore R, Chao A, Jeon S, Faulkner MS, Grey M. Mediators of 12-month outcomes of two internet interventions for youth with type 1 diabetes. J Pediatr Psychol. 2014;39(3):306-315. [doi: 10.1093/jpepsy/jst081] [Medline: 24163439]
12. Kern D, Ljótsson B, Bonnert M, Lindefors N, Kraepelien M. Online guided self-help cognitive behavioral therapy with exposure to anxiety and problem solving in type 1 diabetes mellitus: case study. JMIR Form Res. Jul 13, 2022;6(7):e32950. [FREE Full text] [doi: 10.2196/32950] [Medline: 35830220]
13. Lansing AH, Stanger C, Budney A, Christiano AS, Casella SJ. Pilot study of a web-delivered multicomponent intervention for rural teens with poorly controlled type 1 diabetes. J Diabetes Res. 2016;2016:7485613. [doi: 10.1155/2016/7485613] [Medline: 27610391]
14. Laufer S, Schulze L, Engel S, Klusmann H, Skoluda N, Nater UM, et al. The effect of an internet-based intervention for depression on cortisol and alpha-amylase. Psychoneuroendocrinology. Jun 2023;152:106082. [doi: 10.1016/j.psyneuen.2023.106082] [Medline: 36989562]
15. Liu S, Tanaka R, Barr S, Nolan RP. Effects of self-guided e-counseling on health behaviors and blood pressure: results of a randomized trial. Patient Educ Couns. 2020;103(3):635-641. [doi: 10.1016/j.pec.2019.10.007] [Medline: 31669047]
16. Nolan RP, Feldman R, Dawes M, Kaczorowski J, Lynn H, Barr SI, et al. Randomized controlled trial of e-counseling for hypertension: REACH. Circ Cardiovasc Qual Outcomes. 2018;11(7):e004420. [doi: 10.1161/CIRCOUTCOMES.117.004420] [Medline: 30006474]
17. Park HY, Lee H, Jhee JH, Park KM, Choi EC, An SK, et al. Changes in resting-state brain connectivity following computerized cognitive behavioral therapy for insomnia in dialysis patients: A pilot study. Gen Hosp Psychiatry. 2020;66:24-29. [FREE Full text] [doi: 10.1016/j.genhosppsych.2020.05.013] [Medline: 32615333]
18. Peerani F, Watt M, Ismond KP, Whitlock R, Ambrosio L, Hotte N, et al. A randomized controlled trial of a multicomponent online stress reduction intervention in inflammatory bowel disease. Therap Adv Gastroenterol. 2022;15:17562848221127238. [doi: 10.1177/17562848221127238] [Medline: 36187365]
19. Penedo FJ, Fox RS, Walsh EA, Yanez B, Miller GE, Oswald LB, et al. Effects of web-based cognitive behavioral stress management and health promotion interventions on neuroendocrine and inflammatory markers in men with advanced prostate cancer: a randomized controlled trial. Brain Behav Immun. 2021;95:168-177. [FREE Full text] [doi: 10.1016/j.bbi.2021.03.014] [Medline: 33737170]
20. Romero-Sanchiz P, Nogueira-Arjona R, Araos P, Serrano A, Barrios V, Argente J, et al. Variation in chemokines plasma concentrations in primary care depressed patients associated with internet-based cognitive-behavioral therapy. Sci Rep. 2020;10(1):1078. [doi: 10.1038/s41598-020-57967-y] [Medline: 31974503]
21. Schakel L, Veldhuijzen DS, van Middendorp H, Prins C, Drittij AMHF, Vrieling F, et al. An internet-based psychological intervention with a serious game to improve vitality, psychological and physical condition, and immune function in healthy male adults: randomized controlled trial. J Med Internet Res. Jul 24, 2020;22(7):e14861. [FREE Full text] [doi: 10.2196/14861] [Medline: 32706667]
22. Schumacher S, Engel S, Niemeyer H, Küster A, Burchert S, Skoluda N, et al. Salivary cortisol and alpha-amylase in posttraumatic stress disorder and their potential role in the evaluation of cognitive behavioral treatment outcomes. J Trauma Stress. 2022;35(1):78-89. [doi: 10.1002/jts.22683] [Medline: 34022094]
23. Urech C, Scherer S, Emmenegger M, Gaab J, Tschudin S, Hoesli I, et al. Efficacy of an internet-based cognitive behavioral stress management training in women with idiopathic preterm labor: a randomized controlled intervention study. J Psychosom Res. 2017;103:140-146. [doi: 10.1016/j.jpsychores.2017.10.014] [Medline: 29167041]
24. Webb J, Peerbux S, Ang A, Siddiqui S, Sherwani Y, Ahmed M, et al. Long-term effectiveness of a clinician-assisted digital cognitive behavioral therapy intervention for smoking cessation: secondary outcomes from a randomized controlled trial. Nicotine Tob Res. Oct 26, 2022;24(11):1763-1772. [FREE Full text] [doi: 10.1093/ntr/ntac113] [Medline: 35470860]
